# Editorial Notes

**Published:** 1893-03

**Authors:** 


### Editorial Notes.

DR. THOMAS B. PERRY, of the W. S. Marine Hospital Service, now stationed at Fortress Monroe, Va., paid us a pleasant visit last week. We are always glad to see the doctor, as he is an old Georgia boy.

DR. J. W. BAILEY, of Gainesville, Ga., called on us a few days ago enroute home from Florida, where he had been for a month taking a much needed rest. The Doctor's eminence as a specialist of the diseases of women and children is well known, and we hope to favor our readers with some interesting articles from him in the near future.

WE have received the first issue of the *Woman's Medical Journal*, published at Toledo, O. It is edited by Dr. Margaret L. Hackedorn.

DR. A. A. SMITH, of Hawkinsville, Ga., President of the Medical Association of Georgia, was in the city last week and we were favored with a pleasant call from him. The Doctor informs us that from the present indications the meeting of the Association in Americus, April 19th, 20th and 21st, will be largely attended, and an excellent programme prcsented.

The Association is to be congratulated on having such an able and enthusiastic president.

DR. A. W. CALHOUN has returned home after a months sojourn in Mexico.

WE are in receipt of the first issue of *The New York Polyclinic*, a monthly journal, edited by the professors of the New York Polyclinic, and published by our friend, Dr. Ferdinand King, who became so well known by his admirable management of the *International Journal of Surgery*. We wish the *Polyclinic* unbounded success.

WE had calls from Drs. J. F. Lancaster, of Forsyth, Ga., and J. C. Fields, of Oglethorpe, Ga., last week.

---

DR. NICHOLAS SENN, president of the Rush Medical College of Chicago, and president of the American Co-Medical Association, stopped over in the city for a few hours yesterday and was taken in charge by Dr. J. McF. Gaston, who showed him over the Grady Hospital. Afterwards the doctor delivered a clinical lecture to the students of the Southern Medical College, which was listened to with much interest. The doctor resumed his journey yesterday evening.

---

WE are informed that Dr. Westmoreland will continue his Clinic on Wednesdays, at the Atlanta Medical College, during the coming spring and summer.

